# Making plant methane formation visible—Insights from application of ^13^C‐labeled dimethyl sulfoxide

**DOI:** 10.1002/pei3.10076

**Published:** 2022-05-06

**Authors:** Moritz Schroll, Katharina Lenhart, Steffen Greiner, Frank Keppler

**Affiliations:** ^1^ Institute of Earth Sciences Heidelberg University Heidelberg Germany; ^2^ Bingen University of Applied Sciences Bingen Germany; ^3^ Center for Organismal Studies (COS) Heidelberg Germany; ^4^ Heidelberg Center for the Environment (HCE) Heidelberg University Heidelberg Germany

**Keywords:** dimethyl sulfoxide, light intensity, methane (CH_4_) formation, plants, stable carbon isotopes, stable isotope labelling, vegetation, δ^13^C values

## Abstract

- Methane (CH_4_) formation by vegetation has been studied intensively over the last 15 years. However, reported CH_4_ emissions vary by several orders of magnitude, thus making global estimates difficult. Moreover, the mechanism(s) for CH_4_ formation by plants is (are) largely unknown.
- Here, we introduce a new approach for making CH_4_ formation by plants clearly visible. By application of ^13^C‐labeled dimethyl sulfoxide (DMSO) onto the leaves of tobacco plants (*Nicotiana tabacum*) and Chinese silver grass (*Miscanthus sinensis*) the effect of light and dark conditions on CH_4_ formation of this pathway was examined by monitoring stable carbon isotope ratios of headspace CH_4_ (δ^13^C‐CH_4_ values).
- Both plant species showed increasing headspace δ^13^C‐CH_4_ values while exposed to light. Higher light intensities increased CH_4_ formation rates in *N. tabacum* but decreased rates for *M. sinensis*. In the dark no formation of CH_4_ could be detected for *N. tabacum*, while *M. sinensis* still produced ~50% of CH_4_ compared to that during light exposure.
- Our findings suggest that CH_4_ formation is clearly dependent on light conditions and plant species and thus indicate that DMSO is a potential precursor of vegetative CH_4_. The novel isotope approach has great potential to investigate, at high temporal resolution, physiological, and environmental factors that control pathway‐specific CH_4_ emissions from plants.

## Graphical abstract

Here, we introduce a new approach for making methane (CH_4_) formation by plants clearly visible. By application of ^13^C‐labelled dimethyl sulfoxide (DMSO) onto two different plant species and examing the effect of light and dark conditions on CH_4_ formation of this pathway by monitoring stable carbon isotopes of CH_4_ (δ^13^C‐CH_4_ values). Our findings suggest that CH_4_ formation is clearly dependent on light conditions and plant species and indicate that DMSO is a potential precursor of vegetative CH_4_. This novel isotope approach has great potential to investigate, at high temporal resolution, physiological and environmental factors that control CH_4_ emissions from plants.

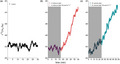

## 1 INTRODUCTION

Nonmicrobial methane (CH_4_) formation from vegetation under aerobic conditions has been controversially debated since its discovery (Beerling et al., 2008; Bloom et al., 2010; Bruhn et al., 2012; Dueck et al., 2007; Evans, 2007; Fraser et al., 2015; Keppler et al., 2006, 2009; Kirschbaum & Walcroft, 2008; Lenhart, Weber, et al., 2015; McLeod et al., 2008; Nisbet et al., 2009; Vigano et al., 2008; Wang et al., 2008). However, even though numerous studies have now confirmed terrestrial plants as a direct source of CH_4_ to the atmosphere, reported emission rates vary by orders of magnitude making global estimates difficult (Bloom et al., 2010; Carmichael et al., 2014; Ferretti et al., 2007; Houweling et al., 2006; Keppler et al., 2006; Kirschbaum et al., 2007; Megonigal & Guenther, 2008; Parsons et al., 2006). Many studies have linked the formation of CH_4_ in plants to environmental and physiological factors such as temperature (Abdulmajeed et al., 2017; Bruhn et al., 2009; Keppler et al., 2009; Martel et al., 2020; Vigano et al., 2008), UV‐irradiation (Keppler et al., 2009; McLeod et al., 2008; Vigano et al., 2008), light (Abdulmajeed et al., 2017; Brüggemann et al., 2009; Martel et al., 2020; Martel & Qaderi, 2017), reactive oxygen species (ROS) (Althoff et al., 2010, 2014; Messenger et al., 2009), and carbon dioxide concentration (Qaderi & Reid, 2011).

Several precursor compounds for CH_4_ formation from living and dead plants have already been identified. These include pectin (Keppler et al., 2009; McLeod et al., 2008; Messenger et al., 2009), lignin (Vigano et al., 2008, 2009), cellulose (Vigano et al., 2008, 2009), ascorbic acid (Althoff et al., 2010), leaf surface waxes (Bruhn et al., 2014), and methionine (Althoff et al., 2014; Han et al., 2017; Lenhart, Althoff, et al., 2015), which contain methyl‐ (‐CH_3_), methoxy‐(‐O‐CH_3_), hydroxymethyl‐(‐CH_2_‐OH), or thiomethyl‐(‐S‐CH_3_) groups, respectively.

Recently CH_4_ produced in plants was suggested to act as a signaling and regulatory molecule playing a physiological role (for more detailed information see review by Wang et al., 2020). It has also been thought that CH_4_ interacting within the metabolism of vegetation plays a role in plant stress response, growth, development, and rooting (Cui et al., 2015; Kou et al., 2018; Qi et al., 2017). Abiotic stress in plants leads to oxidative damage, due to rapid overproduction of ROS (Møller et al., 2007). In order to counterbalance the harmful effects of ROS, plants possess antioxidant enzymes as defense systems. The activity of these antioxidant enzymes has been shown to positively correlate with CH_4_, potentially boosting the plants' defense systems (Cui et al., 2015, 2017; Foyer & Noctor, 2011; Noctor et al., 2012; Uzilday et al., 2014; Zhu et al., 2016).

So far, several pathways of CH_4_ formation in living and dead plants have been identified: (1) UV‐light produced reactive oxygen species (ROS) reacting with methyl groups of plant compounds for example pectin (Bruhn et al., 2009; Keppler et al., 2009; McLeod et al., 2008; Messenger et al., 2009), (2) plant mitochondria in which the electron transport chain was interrupted (Wishkerman et al., 2011), (3) the amino acid methionine in the presence of hydrogen peroxide (H_2_O_2_) and iron salts form methyl radicals, which further react to form CH_4_ or oxidized products such as methanol (Althoff et al., 2014; Benzing et al., 2017; Lenhart, Althoff, et al., 2015), (4) amino acid methyl groups reacting with ROS (Althoff et al., 2014; Martel & Qaderi, 2019). In recent years great progress in the understanding of CH_4_ formation by plants has been made with the identification of organic precursor compounds and the likely important role of methyl radical formation (Althoff et al., 2014; Messenger et al., 2009). Recently Ernst et al. (2022) proposed a reaction mechanism for CH_4_ formation that might occur on a cellular level across all living organisms. Thereby, CH_4_ is formed by the interaction of ROS with free iron and methylated sulfur and nitrogen compounds in living cells. Furthermore, the authors showed that increasing the level of oxidative stress enhanced CH_4_ production in all of the investigated organisms. However, it is clear that there are many physiological and environmental variables that likely control emissions. Particularly, under field‐like conditions, an enormous experimental and analytical effort has to be made to determine CH_4_ emission rates from specific organisms to the atmosphere but also to identify the environmental factors that regulate their emissions (Wang et al., 2020). Consequently, the detection and quantification of plant CH_4_ emission rates remain challenging tasks not only because of the lack of knowledge about mechanism(s) and effects of environmental factors on CH_4_ formation, but also due to the need for highly sensitive and appropriate experimental facilities, as changes in CH_4_ mixing ratios during incubation experiments are often only in the ppbv range (see e.g., Brüggemann et al., 2009; Keppler et al., 2006). In order to overcome many of the analytical problems and to gain deeper insights into the complex processes involved in plant CH_4_ emissions, stable isotope labelling techniques are now commonly used (Brüggemann et al., 2009; Keppler et al., 2006; Lenhart, Weber, et al., 2015 , Weber, et al., 2015). Hence, in the recent past, stable carbon isotope labeled compounds, such as ^13^C‐CO_2_ and ^13^C‐methionine, have already been used as a tool to demonstrate CH_4_ emissions by vegetation. When conducting stable carbon isotope labelling experiments, methionine was established as a precursor of CH_4_ release by plants and emission rates were shown to be controlled by physical stress factors (Lenhart, Althoff, et al., 2015). However, the addition of methionine to plant systems is accomplished through assimilation via the roots and thus the actual amount of absorbed methionine is hard to assess. In contrast, dimethyl sulfoxide (DMSO) can be applied to the plant leaves and due to its physiochemical properties, it penetrates the leaf surface and enters the plant system almost instantly (Anchordoguy et al., 1992; Gurtovenko & Anwar, 2007). DMSO has been used as an indicator for the production of hydroxyl radicals (·OH) in biological systems by the formation of CH_4_ (Klein et al., 1981; Repine et al., 1981). Here, a methyl radical is cleaved from DMSO during reaction with ·OH (or other ROS) eventually leading to the formation of CH_4_ by abstraction of hydrogen from other available organic molecules. In plants DMSO leads to increased H_2_O_2_ concentrations (Mannan et al., 2010) and was linked to plant parts experiencing oxidative stress, such as yellowing and senescing leaves, indicating its antioxidant effects (Husband & Kiene, 2007).

In this study, we tested an isotopic approach to gain further insight into the various environmental factors that control CH_4_ emissions from plants. Particular focus was put on the role of light–dark cycles and light intensity on CH_4_ formation from plants and if these factors can be made visible by applying isotopically labeled DMSO as a CH_4_ precursor. Therefore, we applied ^13^C‐labeled DMSO onto the leaves of tobacco plants (*Nicotiana tabacum*) and Chinese silver grass (*Miscanthus sinensis*), which had been cultivated under sterile conditions, and monitored both CH_4_ mixing ratios and δ^13^C‐CH_4_ values during static and dynamic chamber incubation experiments. For high‐resolution measurements of CH_4_ mixing ratios and δ^13^C‐CH_4_ values during dynamic experiments, we employed Cavity Ring‐Down Spectroscopy (CRDS), while for static incubation experiments, gas samples were withdrawn and measured by isotope ratio mass spectrometry (IRMS).

## 2 MATERIALS AND METHODS

### 2.1 Selected plants

*Nicotiana tabacum* and *Miscanthus sinensis* were cultured under sterile conditions for our investigations. Both plant species were chosen for this study as there was already considerable in‐house practical experience in the sterile incubation of these species. Moreover, these are plants with a distant phylogenetic relationship (dicotyledonous vs monocotyledonous plants). They differ significantly not only morphologically and anatomically, but also physiologically, for example, in their type of photosynthesis, as *N. tabacum* is a C_3_‐plant and *M. sinensis* is a C_4_‐plant. This physiological disparity causes different responses to varying parameters such as CO_2_ concentration, light intensity, or temperature.

### 2.2 Culture of plants under sterile conditions and application of DMSO

Cultivation of *N. tabacum* and *M. sinensis* was achieved following the approach of Lenhart et al. (2019). Both cultures were then incubated with a 16 h daylight phase (light intensity: 150 μmol m^−2^ s^−1^; temperature: 23°C) and an 8 h dark phase (temperature: 23°C). The constant temperature of 23°C throughout our experiments was chosen to prevent the potential influence of temperature changes on our results, making light intensity the only changing variable. Measurements on the cultures were conducted once the shoots grew to ca. 10 cm (see Table S1 for plant characteristics). By this stage, the cuttings of *N. tabacum* were fully rooted, while the *M. sinensis* shoots did not develop roots under these conditions. In order to determine the optimal amount of DMSO to apply to the plant leaves so that no visible impairment to the plant was observed pre‐tests were run with varying DMSO concentrations. Therefore, aqueous solutions of DMSO with concentrations between 5% and 20% were applied to the plant leaves. The plants treated with 5% DMSO solution showed no visible impact, however higher concentrated DMSO solutions led to visible yellowing and damage to the plants (see Figure S1a–c). Hence, an aqueous solution of 5% DMSO was used for our investigations. For incubation experiments of plant cultures, 0.8 ml of a ^13^C‐labeled DMSO solution was applied onto the leaves of the plants using a calibrated pump dispenser. The solution consisted of an aquatic solution of 5% DMSO, whereby 6% of the added DMSO was ^13^C‐labeled and 0.25% of the surfactant Silwet®. Considerable care was taken to ensure that the DMSO solution was uniformly applied to the plant leaves.

### 2.3 Dynamic incubation experiments

**FIGURE 1:**
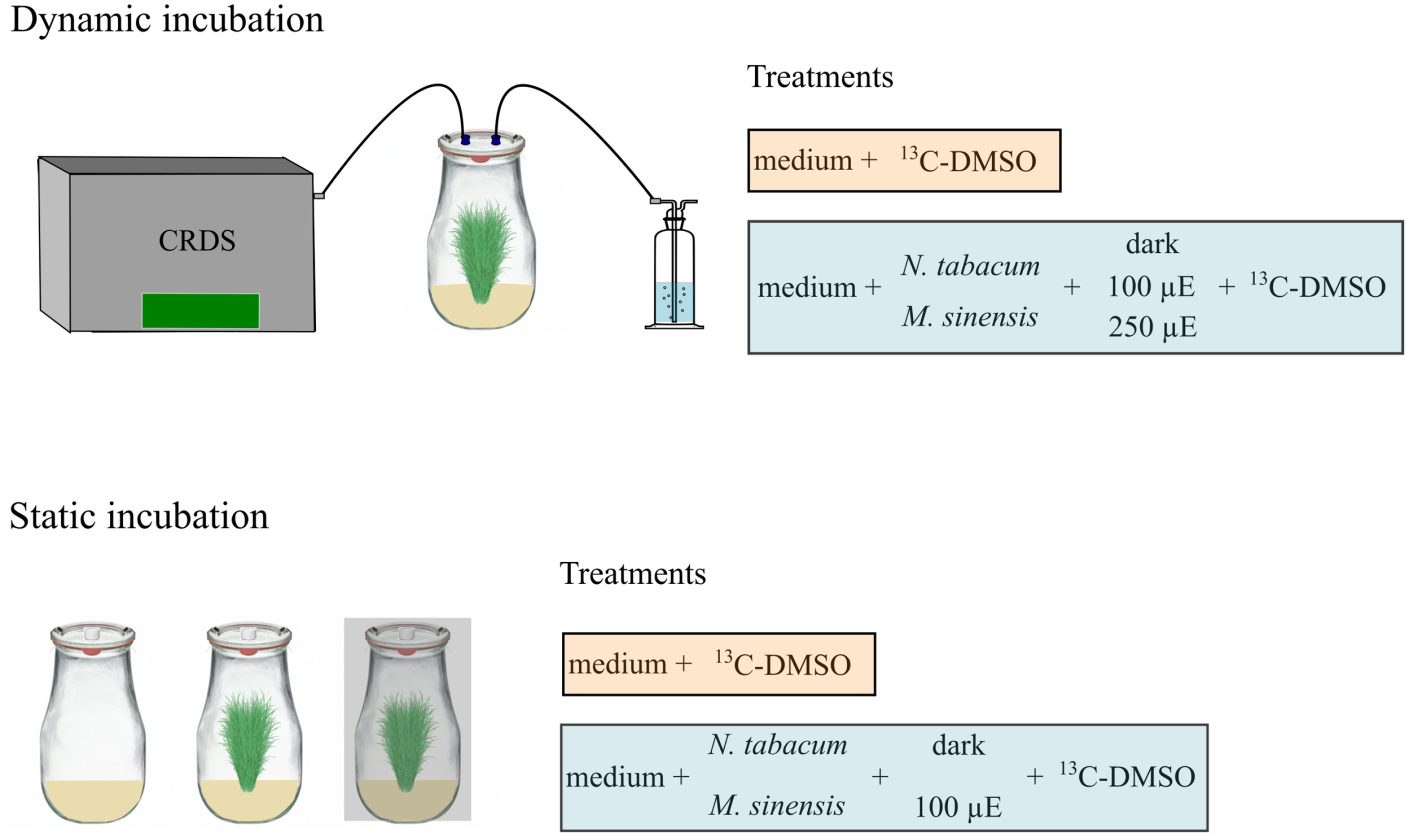
Experimental setup and treatments conducted during dynamic (top) and static (bottom) incubations

The experimental setup used for dynamic incubations is illustrated in Figure 1 (top). All experiments took place in a climate chamber at 23°C in the dark and light at intensities of either 100 or 250 μmol m^−2^ s^−1^ (light intensity measured inside the glass flasks). A cavity ringdown spectroscope (CRDS, Picarro G2201‐i) was coupled to the 2.7 L glass flasks containing the sterile cultures of *N. tabacum* and *M. sinensis* via an IQS plug connector (6 mm inner diameter) (see Figure S2b). The CRDS enabled simultaneous measurement of headspace CH_4_ mixing ratios and δ^13^C‐CH_4_ values of the headspace air inside the glass flasks with an inflow rate of 23 ml min^−1^. In order to avoid low‐pressure conditions during the measurements, which might influence the well‐being and metabolism of the plants, ambient air was allowed to flow into the glass flasks after first passing through a gas washing bottle (volume: 25 ml; deionized water volume: 15 ml). This was to ensure a consistent humidity of the inflowing ambient air and therefore to keep environmental parameters constant to minimize any effects on the plants during their incubation, for example, to prevent the plants from closing their stomata. Plant species were incubated in the dark (*n* = 2) and light at intensities of either 100 or 250 μmol m^−2^ s^−1^ (*n* = 3–4) for time periods varying between 0.5 and 14 h. In one experiment, incubation in the dark (max. 0.5 h) was immediately followed by incubation in light (max. 0.5 h). While in another experiment both plant species were examined during a light (1–2 h)–dark (10 h)–light (2–3 h) incubation cycle. Both parts of the dynamic incubation experiment were designed to examine the effect of light on the change of δ^13^C‐CH_4_ values in the headspace gas of incubation flasks, hence indicating the formation of ^13^C‐CH_4_ from ^13^C‐labeled DMSO. To allow comparison between the different treatments and individual incubations and to account for the different incubation periods headspace δ^13^C‐CH_4_ change rates were calculated using a simple linear regression analysis of the 2‐min average sample points. Additionally, a simple dilution model was employed in order to correct headspace δ^13^C‐CH_4_ change rates for the dilution with laboratory air during dynamic incubation (see Methods S1, Figure S3). Hence, δ^13^C‐CH_4_ values corrected by the dilution model are reported.

### 2.4 Static incubation experiments

The experimental setup used for static incubations is illustrated in Figure 1 (bottom). Cultures of *N. tabacum* and *M. sinensis* were incubated in 2.7 L glass flasks (see Figure S2a,c,d). The glass flasks were closed with glass lids, containing a hole were fitted with silicone stoppers (Saint‐Gobain Performance Plastics) to allow headspace sampling of the plant cultures. Both plant species were incubated under dark and light conditions (intensity of 100 μmol m^−2^ s^−1^ inside the glass flasks). All treatments for the static incubation experiment were performed in triplicate (*n* = 3) and samples were taken after 1, 4, 9, and 21 h. Headspace samples were collected using 60 ml PE syringes (Plastikpak, BD) and transferred to evacuated 12 ml exetainers (Labco). At every sampling time, 40 ml were removed for CH_4_ mixing ratio measurements and another 40 ml for δ^13^C‐CH_4_ stable isotope measurements. Directly after sampling air with an equivalent volume of withdrawn headspace gas was added to the flasks to avoid pressure changes. The mixing ratio of CH_4_ and δ^13^C‐CH_4_ values were corrected to account for this dilution.

### 2.5 Definition of δ values

Stable carbon isotope values are given in the conventional “δ (delta) notation” in per mil versus Vienna Peedee Belemnite (V‐PDB). The δ notation is defined as the relative difference in the isotope ratio of a substance compared to the standard substance Vienna Peedee Belemnite (see Equation 1). δ^13^C‐CH_4_ values are expressed in ‰ versus V‐PDB throughout the whole of this manuscript.

(1) δC13=C13C12sampleC13C12V−PDB–1

### 2.6 Analysis of δ ^13^C‐CH_4_ values and CH_4_ mixing ratios in dynamic experiments

Headspace CH_4_ mixing ratios and δ^13^C‐CH_4_ values during the dynamic incubation experiment were measured using a Picarro G2201‐i CRDS. As described above the CRDS was directly connected to the incubation glass flasks with a flow rate of 23 ml min^−1^ and measurements were made with a very high temporal resolution of 1 Hz. Before measurement, the headspace gas was passed through a Nafion drying tube (Nafion MD110, PermaPure LLC) in order to remove water from the sample and thus avoid optical interferences during the measurement of CH_4_. Thus, we determined dry mole fractions of CH_4_ which we simply express as mixing ratio. The guaranteed CH_4_ mixing ratio precision of the manufacturer was 5 ppbv + 0.05% of reading, while the given precision for δ^13^C‐CH_4_ values is below 0.8‰. In order to rule out instrument drift during measurements with the CRDS compressed air (stored in 1 L Tedlar gas sampling bags) was measured before and after the incubation experiment. The measured drift controls showed no difference for the CH_4_ mixing ratios and only minor differences (0.3%) for δ^13^C‐CH_4_ values. Hartmann (2018), showed that there is a difference between δ^13^C‐CH_4_ values measured with our CRDS and those with a continuous flow mass spectrometry system (CF‐IRMS, see Section 2.7 below). Therefore, reported δ^13^C‐CH_4_ values were corrected according to Hartmann (2018).

### 2.7 Analysis of δ ^13^ C‐CH_4_ values and CH_4_ mixing ratios in static experiments

Headspace samples collected from the static incubation experiments were analyzed for their CH_4_ mixing ratios by a gas chromatograph (GC, Bruker Greenhous Gas Analyzer 450‐GC) equipped with a flame ionization detector (FID). Before entering the analytical system, the gas sample was passed through a chemical trap filled with Drierite® to remove water. For calibration of the GC‐FID five reference gases (Deuste Gas Solutions GmbH), ranging from 1 ppmv (parts per million by volume) to 21 ppmv were used. Peaks were integrated using Galaxie software (Varian Inc.).

δ^13^C‐CH_4_ values of headspace gas samples collected from static incubation experiments were measured using a continuous flow isotoperatio mass spectrometer (CF‐IRMS) according to the method described in Schroll et al. (2020) (see Methods S2) and sample values were normalized using the method of Paul et al. (2007).

### 2.8 Calculation of headspace CH_4_ produced from ^13^ C‐labeled DMSO

The amount of CH_4_ produced from ^13^C‐labeled DMSO was calculated via a simple mass balance approach (Equation 2):

(2) $$c{\left({\mathrm{CH}}_{4}\right)}_{b}*i{\left({\mathrm{CH}}_{4}\right)}_{b}+c{\left({\mathrm{CH}}_{4}\right)}_{p}*i{\left({\mathrm{CH}}_{4}\right)}_{p}=c{\left({\mathrm{CH}}_{4}\right)}_{t}*i{\left({\mathrm{CH}}_{4}\right)}_{t}$$

where c(CH_4_)_b_, c(CH_4_)_p,_ c(CH_4_)_t_ are the mixing ratios of background CH_4_, CH_4_ produced during incubation and total CH_4_ and i(CH_4_)_b_, i(CH_4_)_p,_ i(CH_4_)_t_ are the isotopic compositions of background CH_4_, CH_4_ produced during incubation and total CH_4_, respectively. With the assumption from Equation 3 and the insertion of Equation 3 into Equation 2, Equation 4 was used to calculate the amount of headspace CH_4_ formed from ^13^C‐labeled DMSO:

(3) $$c{\left({\mathrm{CH}}_{4}\right)}_{t}=c{\left({\mathrm{CH}}_{4}\right)}_{p}+c{\left({\mathrm{CH}}_{4}\right)}_{b}$$

(4) $$c{\left({\mathrm{CH}}_{4}\right)}_{p}=\frac{\left(c{\left({\mathrm{CH}}_{4}\right)}_{b}*i{\left({\mathrm{CH}}_{4}\right)}_{t}\right)-\left(c{\left({\mathrm{CH}}_{4}\right)}_{b}*i{\left({\mathrm{CH}}_{4}\right)}_{b}\right)}{i{\left({\mathrm{CH}}_{4}\right)}_{p}-i{\left({\mathrm{CH}}_{4}\right)}_{t}}$$

### 2.9 Statistics

The rates of δ^13^C‐CH_4_ change in permil per hour for both the dynamic and static incubation experiments are presented as the arithmetic means of the respective replicates together with their standard deviation (SD). Arithmetic means and SDs were calculated using Microsoft Excel (Microsoft Excel for Office 365 MSO). Linear regression analysis was conducted using the software R version 4.0.3 (R Core Team, 2020) with the package “tidyverse” (Wickham et al., 2019). For the evaluation of the dataset of this study, various statistical tests were conducted (SigmaPlot 12.2.0.45). Please note that the term “statistically significant” (p < *0.05*) was avoided in this study, following the recommendations of the American Statistical Association (ASA) on *p*‐values and statistical significance. Thus, for the analysis, as well as, conclusions and scientific rationale of this dataset, statistical indicators were applied but not used as the exclusive criteria (Wasserstein et al., 2019).

## 3 RESULTS

We first present the results for headspace CH_4_ mixing ratio measurements and then show the stable carbon isotope values of headspace CH_4_ separately for the dynamic and static incubation experiments of the plant species *N. tabacum* and *M. sinensis* after the application of ^13^C‐labeled DSMO. For both the dynamic and static incubations we first show results of the effect of light–dark cycles and thereafter the influence of light intensity on the change of headspace δ^13^C‐CH_4_ values in the incubation flasks. Finally, we compare the headspace δ^13^C‐CH_4_ change rates for each treatment, both incubation types and the differences between the two plant species.

### 3.1 Changes in CH_4_ mixing ratio during incubation in static and dynamic chambers

**FIGURE 2:**
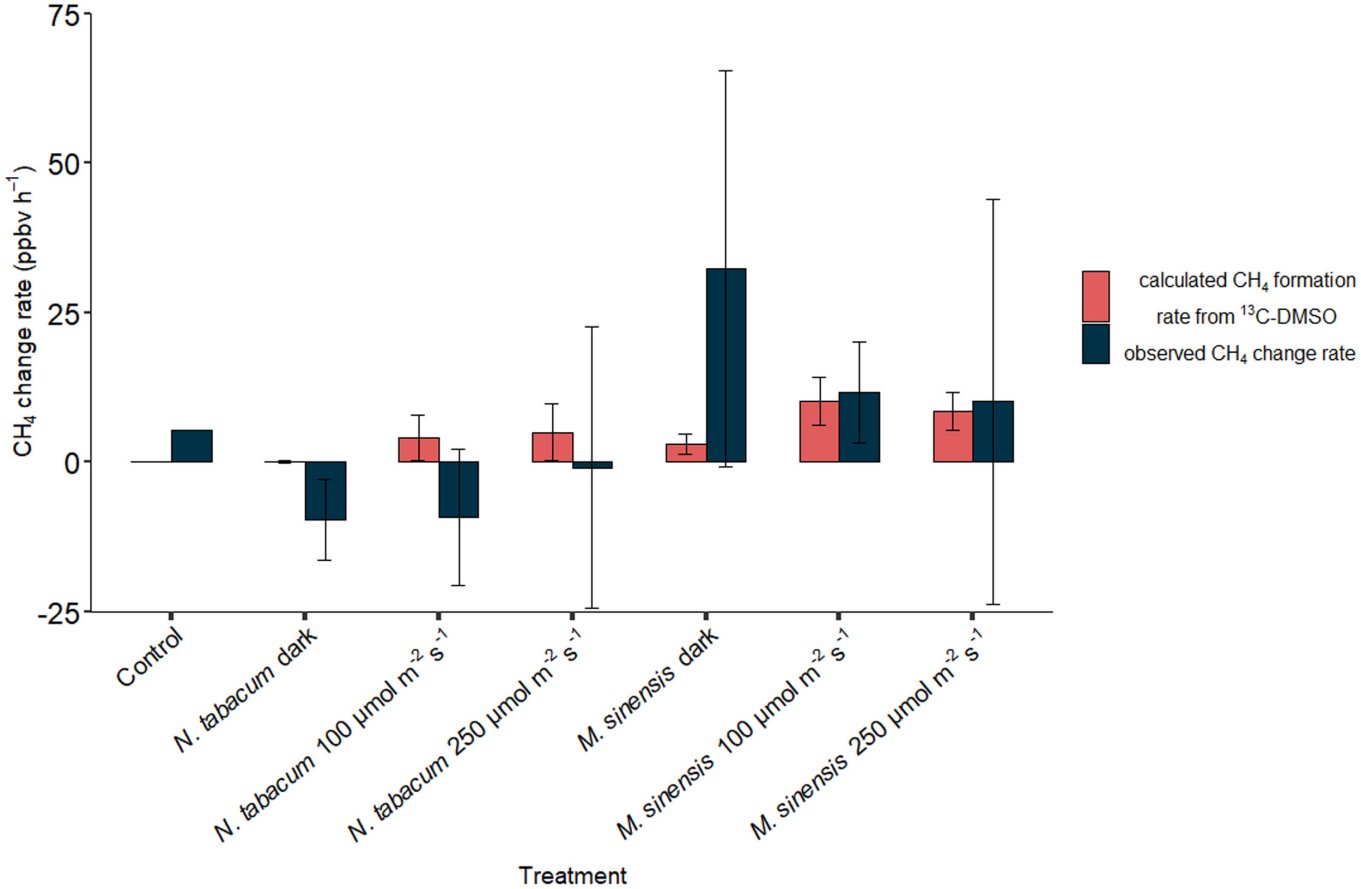
Calculated CH_4_ formation rates from ^**13**^C‐labeled DMSO (red) and observed change in CH_4_ mixing ratios (blue) as mean values for controls (*n* = 2) and mean values and SDs for *Nicotiana tabacum* (*n* = 3–4) and *Miscanthus sinensis* (*n* = 3–4) samples during dynamic incubation under different light conditions

The measured total range of observed changes in headspace CH_4_ mixing ratios (change rate provided in ppbv h^−1^) during incubation of *N. tabacum* and *M. sinensis* samples (with added ^13^C‐labeled DSMO) and controls (medium with added ^13^C‐labeled DSMO) ranged from −9 ± 11 to 32 ± 33 ppbv h^−1^ (Figure 2). For comparison, Figure 2 shows the results of the calculated CH_4_ change rates derived from the observed changes in headspace δ^13^C‐CH_4_ values of the plants when ^13^C‐labeled DMSO was applied (see isotopic sections below, Table S2 and Equation 4). Methane formation rates solely derived from DMSO range between 0 and 0.4 ppbv h^−1^ for controls, 0.1 ± 0.2, 4.1 ± 3.8, and 5.0 ± 4.7 ppbv h^−1^ for *N. tabacum* and 3.1 ± 1.6, 10.2 ± 4.0, and 8.5 ± 3.2 ppbv h^−1^ for *M. sinensis* incubated in the dark, and light at 100 and 250 μmol m^−2^ s^−1^, respectively (Figure 2). Thus, maximum CH_4_ formation rates calculated from the application of ^13^C‐labeled DMSO lie within SDs of the measured changes of CH_4_ mixing ratios.

For static incubations, the observed change rate in CH_4_ mixing ratios was in the range of the SD of the triplicate measurements, but as SDs were high (up to 2 ppbv h^−1^) no clear trend could be detected for any of the treatments (see Figure S4). The calculated formation rate of CH_4_ derived from DMSO was in the range of 0.04 ± 0.02 for controls, 0.08 ± 0.01 and 1.8 ± 0.2 ppbv h^−1^ for *N. tabacum* and 1.0 ± 0.4 and 1.8 ± 0.4 ppbv h^−1^ for *M. sinensis* during incubation in the dark and under light intensity 100 μmol m^−2^ s^−1^, respectively. Maximum CH_4_ formation from DMSO, therefore, lay within SDs of the individual triplicate measurements.

In both the dynamic and static incubation experiments the influence of dark–light cycles and light intensity on CH_4_ formation were difficult to clearly establish by measurement of mixing ratios because changes lay within the range of the observed SDs. Therefore, the influence of these factors on the release of CH_4_ by the plant species were further assessed by the release of ^13^C‐CH_4_ after the application of ^13^C‐labeled DMSO, as changes in the resulting δ^13^C‐CH_4_ values might be much better resolved than those monitored by mixing ratio measurements.

### 3.2 Dynamic incubation experiment

#### 3.2.1 Effect of light–dark cycles

**FIGURE 3:**
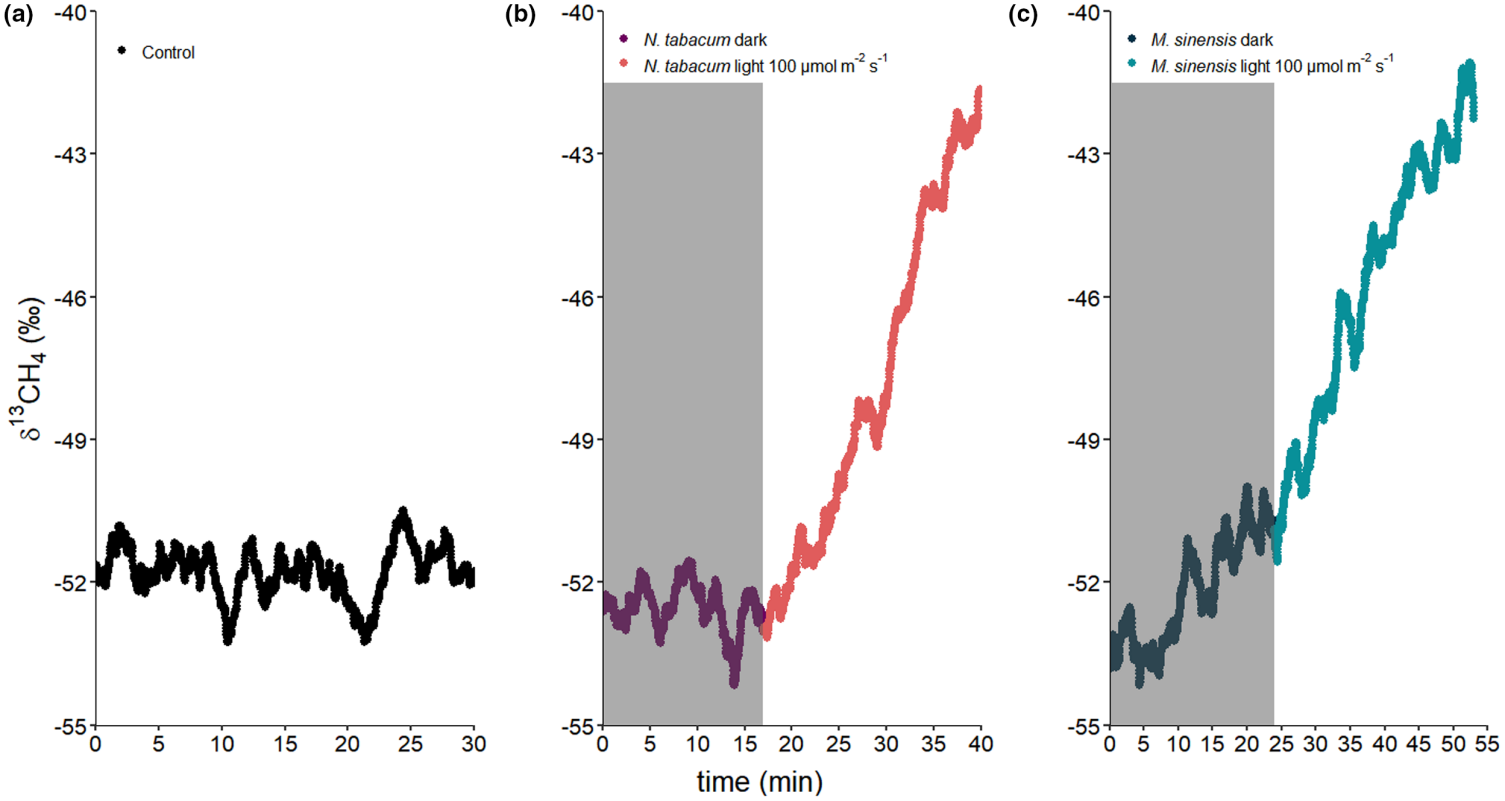
Typical δ^13^C‐CH_4_ values of headspace gas during dynamic incubations of (a) control, (b) *Nicotiana tabacum,* and (c) *Miscanthus sinensis* under dark and light conditions. The dark period is indicated by a gray box. The light intensity during the light cycle was 100 μmol m^−2^ s^−1^. δ^13^C‐CH_4_ values are presented as 2‐min moving average values. ^13^C‐labeled dimethyl sulfoxide (DMSO) was applied to plant leaves before incubation

In the dynamic incubation experiment, the effect of dark and light conditions on δ^13^C values of CH_4_ released by *N. tabacum* and *M. sinensis* was examined following the application of ^13^C‐labeled DMSO (see Section 2.2). Figures 3a–c show typical examples of the trends of the δ^13^C‐CH_4_ values measured for control (medium+^13^C‐labeled DMSO), *N. tabacum* and *M. sinensis*, when the plant species were incubated first in the dark (max. 0.5 h) and then in light at 100 μmol m^−2^ s^−1^ (max. 0.5 h). Controls showed a constant δ^13^C‐CH_4_ value of −51.8 ± 0.5‰ (minimum and maximum δ^13^C‐CH_4_ values of −53.3‰ and −50.5‰, respectively). δ^13^C‐CH_4_ values for emissions from *N. tabacum* incubations in the dark showed little change from those of background noise but increased immediately and steadily after exposure to light from −52.6‰ to −38.4‰. Hence the δ^13^C‐CH_4_ values shifted to more positive values by up to 12.0‰ during the incubation period. In contrast, δ^13^C‐CH_4_ values during incubation of *M. sinensis* in the dark increased by up to 6.0‰ (depending on the δ^13^C‐CH_4_ value at the start of incubation), and then continued to increase at a faster rate immediately after exposure to light, leading to a rise in δ^13^C‐CH_4_ values between 3.6‰ and 15.5‰ at the end of the incubation period.

**FIGURE 4:**
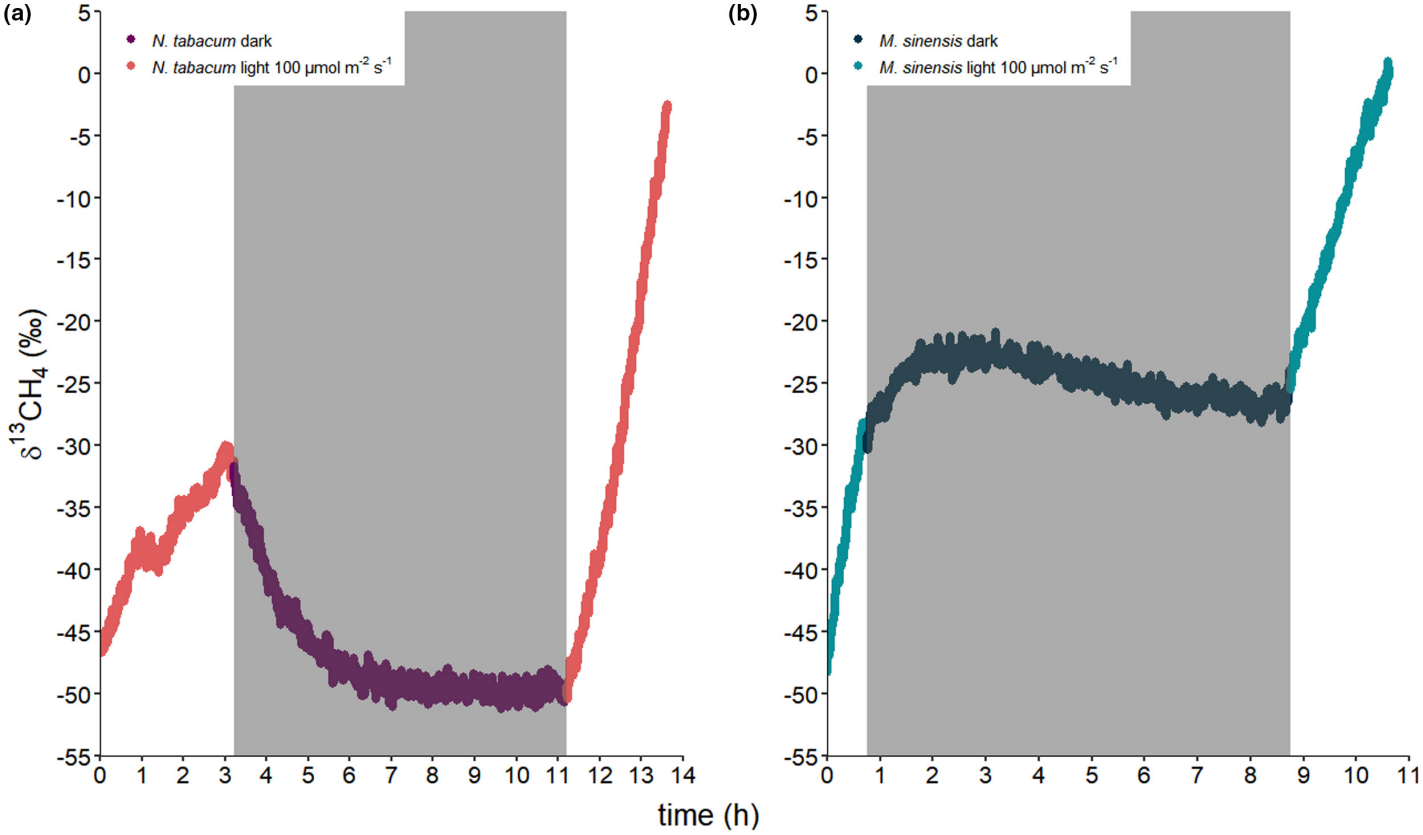
Headspace δ^13^C‐CH_4_ values measured during dynamic incubation of (a) *Nicotiana tabacum* and (b) *Miscanthus sinensis* plants under light–dark–light cycle conditions following application of ^13^C‐labeled dimethyl sulfoxide (DMSO) to their leaves. The dark time period is indicated by a gray box and light intensity was 100 μmol m^−**2**^ s^−**1**^. δ^13^C‐CH_4_ values are presented as 2‐min moving average values

Figure 4 displays the δ^13^C‐CH_4_ headspace values measured during longer incubation periods (up to 14 h) under both dark and light conditions for *N. tabacum* and *M. sinensis*. During the light–dark–light incubation of *N. tabacum* a clear increase in δ^13^C‐CH_4_ values was observed during the first light period (intensity at 100 μmol m^−2^ s^−1^) with a shift from −46.7‰ to −30.0‰. In contrast, during the dark incubation period, δ^13^C‐CH_4_ values decreased toward the atmospheric background value of −51.8‰ but then started to increase again immediately after exposure to light and continued to steadily rise from −50.5‰ to −2.6‰ during this incubation period. Similarly, for *M. sinensis* incubated under the same conditions, a clear increase in δ^13^C‐CH_4_ values was observed during both the first and second period of incubation under light (100 μmol m^−2^ s^−1^) with increases from −48.3‰ to −28.1‰ and −25.5‰ to +1.0‰, respectively. However, interestingly, during the first 50 min of incubation in the dark δ^13^C‐CH_4_ values still increased slightly and then transitioned into a steady decrease in δ^13^C‐CH_4_ values for the remainder of the dark incubation period.

#### 3.2.2 Effect of light intensity

**FIGURE 5:**
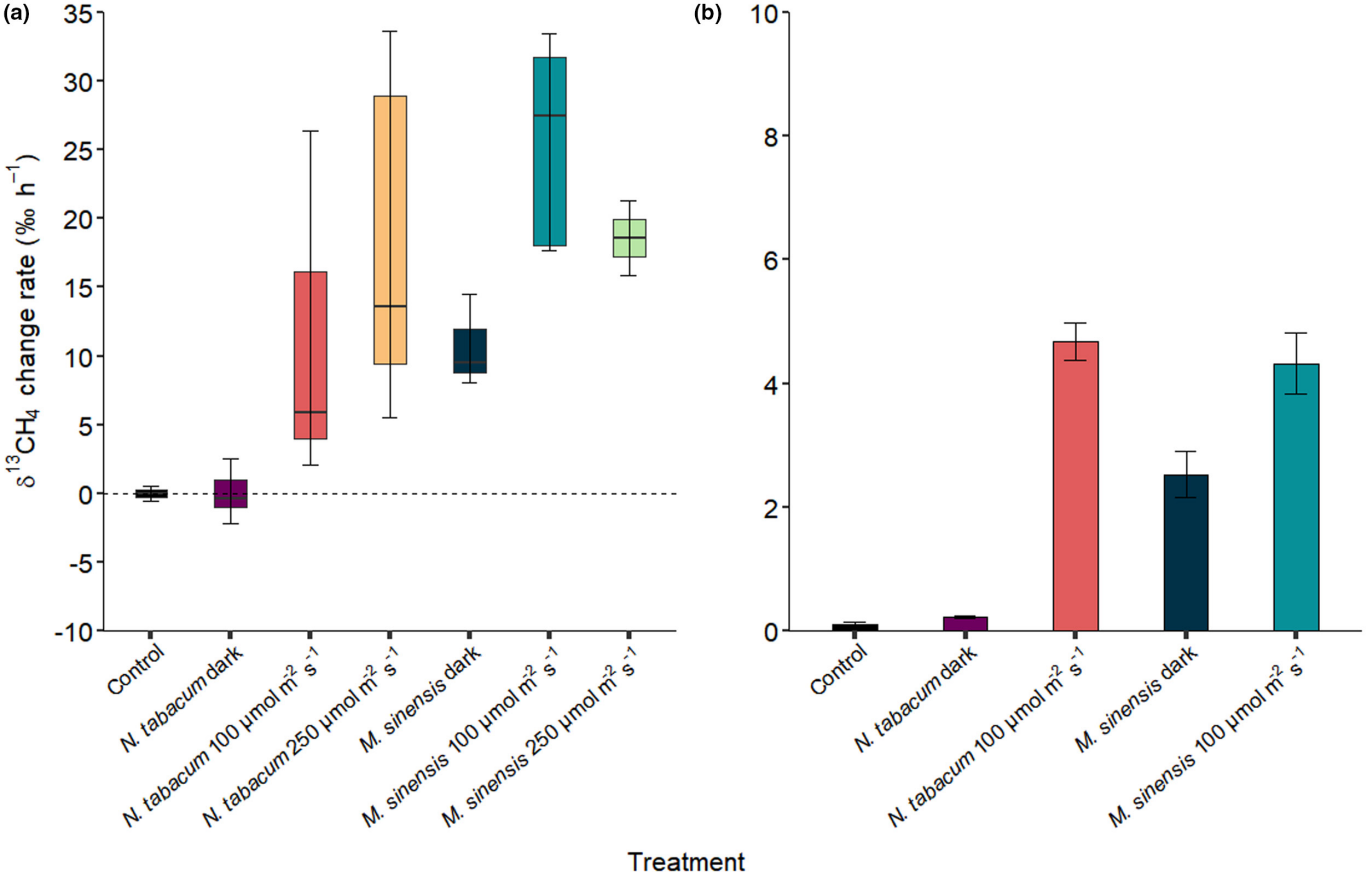
Rates of change of headspace δ^13^C‐CH_4_ values during (a) dynamic and (b) static incubation of *Nicotiana tabacum* and *Miscanthus sinensis* plants under differing light conditions following application of ^13^C‐labeled dimethyl sulfoxide (DMSO) to their leaves. Rates of δ^13^C‐CH_4_ changes are presented as box plots with outliers (*n* = 2–5) and mean values with SD (*n* = 3) for dynamic and static incubations, respectively

The influence of light intensity on the formation of ^13^C‐CH_4_ during the incubation of both plant species was examined at light intensities of 100 and 250 μmol m^−2^ s^−1^ (see Figure 5a). Headspace gas above *N. tabacum* when incubated under light with an intensity of 100 μmol m^−2^ s^−1^ showed an increase in δ^13^C‐CH_4_ values starting from between −54.5‰ and −50.9‰ and reaching between −50.7‰ and −38.5‰ at the end of the incubation period. On average, δ^13^C‐CH_4_ values increased by 7.0 ± 3.8 ‰ (*n* = 3). When *N. tabacum* was incubated under a higher light intensity of 250 μmol m^−2^ s^−1^ headspace δ^13^C‐CH_4_ values increased initially from between −54.2‰ and −52.2‰ to −52.6‰ and −41.6‰ at the end of the incubation period, with an average increase of 5.5 ± 3.9 ‰ (*n* = 4). Headspace δ^13^C‐CH_4_ values for *M. sinensis* incubation under light with an intensity of 100 μmol m^−2^ s^−1^ increased from between −52.1‰ and −47.7‰ to between −44.2‰ and −36.5‰, showing on average an increase of 9.7 ± 2.1 ‰ (*n* = 4). When *M. sinensis* was incubated under light with an intensity of 250 μmol m^−2^ s^−1^ headspace δ^13^C‐CH_4_ values increased from between −49.6‰ and −46.4‰ to between −42.8‰ and −32.8‰, increasing on average by 8.9 ± 4.9‰ (*n* = 3).

### 3.3 Static incubation experiment––Effect of light

**FIGURE 6:**
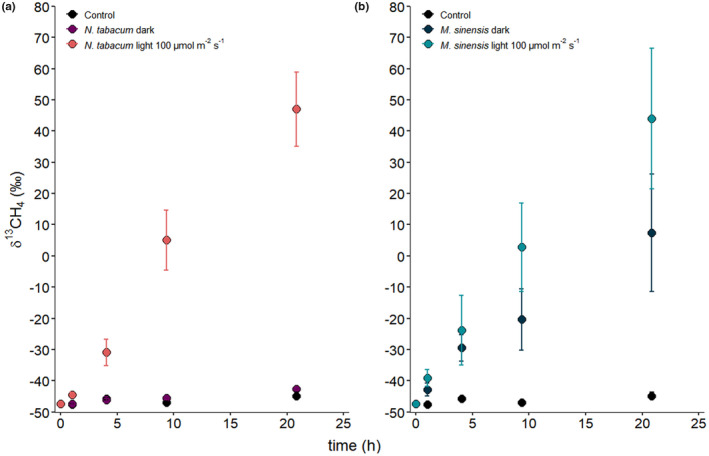
Headspace δ^13^C‐CH_4_ values measured during static incubation of (a) *Nicotiana tabacum* and (b) *Miscanthus sinensis* plants under dark and light (100 μmol m^−**2**^ s^−**1**^) conditions following application of ^**13**^C‐labeled dimethyl sulfoxide (DMSO) to their leaves. Data shows mean values with SD (*n* = 3). Where no error bars are visible, they lie within the data point symbol

In addition to the dynamic light–dark experiments, the effect of exposure to light on headspace δ^13^C‐CH_4_ values was also investigated when both *N. tabacum* and *M. sinensis* were incubated under static conditions (see Sections 2.2 and 2.3). The results from control, *N. tabacum* and *M. sinensis* static incubations are presented in Figure 6. Headspace from control samples showed only a marginal trend toward more positive δ^13^C‐CH_4_ values (−47.3 ± 0.3‰ to −44.8 ± 1.1‰) during the incubation period while considerable change in δ^13^C‐CH_4_ values (−47.3 ± 0.3‰ to +47.1 ± 11.9‰) was observed for *N. tabacum* incubated under light conditions (100 μmol m^−2^ s^−1^). When *N. tabacum* was incubated in the dark only a small shift toward more positive δ^13^C‐CH_4_ values (δ ^13^C‐CH_4_ value of −42.7 ± 0.4‰) was observed. Similar to *N. tabacum*, under light conditions *M. sinensis* showed headspace δ^13^C‐CH_4_ value changes from −47.3 ± 0.3‰ to +44.0 ± 22.5‰. However, during incubation in the dark, and in contrast to *N. tabacum*, headspace δ^13^C‐CH_4_ values for *M. sinensis* still increased substantially from −47.3 ± 0.3‰ to +7.4 ± 18.8‰.

### 3.4 δ^13^ C‐CH_4_ change rates during dynamic and static incubation

The calculated rates of change in headspace δ^13^C‐CH_4_ values for dynamic incubation experiments are displayed in Figure 5a. During incubation in the dark and at both light intensities *N. tabacum* and *M. sinensis* showed a clear trend toward more positive δ^13^C‐CH_4_ change rate values when ^13^C‐labeled DMSO was applied to plant leaves, except for *N. tabacum* when incubated in the dark. Control samples showed only marginal δ^13^C‐CH_4_ change rate values in the range of −0.08 ± 0.09‰ h^−1^ during the incubation period. No notable shift was apparent in the δ^13^C‐CH_4_ change rates calculated for *N. tabacum* with values of 0.0 ± 1.6‰ h^−1^ (*n* = 5, *p* = 0.324 compared to control) when incubated in the dark. However, for this species clear increases in δ^13^C‐CH_4_ rate values were observed during incubation under light conditions with values of 11.5 ± 10.7 (*n* = 5, *p* = 0.087 compared to *N. tabacum* in the dark) and 15.4 ± 11.9‰ h^−1^ (*n* = 3, *p* = 0.001 compared to *N. tabacum* in the dark) at light intensities of 100 and 250 μmol m^−2^ s^−1^ (*p* = 0.246 between both light intensities), respectively. Contrastingly, *M. sinensis* showed δ^13^C‐CH_4_ change rates with values of 10.4 ± 5.0‰ h^−1^ (*n* = 4, *p* = 0.024 compared to control) during incubation in the dark, which accounts for about 40% when compared to δ^13^C‐CH_4_ change rates during incubation under light with 25.7 ± 6.7 (*n* = 5, *p* = 0.018 compared to *M. sinensis* in the dark) and 24.5 ± 8.6‰ h^−1^ (*n* = 3, *p* = 0.098 compared to *M. sinensis* in the dark) at light intensities of 100 μmol m^−2^ s^−1^ and 250 μmol m^−2^ s^−1^ (*p* = 0.853 between both light intensities), respectively. Even though visual differences in the δ^13^C‐CH_4_ change rates between light intensities for both plant species were apparent, as *N. tabacum* showed slightly higher δ^13^C‐CH_4_ change rates at the lower light intensity (*p* = 0.092) and M. sinensis showed lower δ^13^C‐CH_4_ change rates at the higher light intensity (*p* = 0.495), these differences did not express statistically. It is important to point out that any comparison between the δ^13^C‐CH_4_ change rates for the two plant species should be treated with much care as their dry weight and leaf surface area differ substantially and both parameters might likely influence these rates significantly.

In the static incubation experiment (Figure 5b) the calculated δ^13^C‐CH_4_ change rates are generally lower when compared to those from the dynamic incubation experiment. However, the observed pattern and extent of δ^13^C‐CH_4_ change rates was analogous to that of the dynamic incubation experiment. While δ^13^C‐CH_4_ change rates for the control and *N. tabacum* incubated in the dark are small with 0.1 ± 0.03‰ h^−1^ (*n* = 3) and 0.2 ± 0.02‰ h^−1^ (*n* = 3, *p* = 0.088 compared to control), respectively, the highest δ^13^C‐CH_4_ change rate was calculated for *N. tabacum* incubated under light conditions (100 μmol m^−2^ s^−1^) with a rate of 4.7 ± 0.3‰ h^−1^ (*n* = 3, *p* < 0.001 compared to *N. tabacum* in the dark). *M. sinensis* showed a δ^13^C‐CH_4_ change rate of 2.5 ± 0.4‰ h^−1^ (*n* = 3, *p* = 0.020 compared to control), which is similar to the dynamic incubation experiment, and would account for about 60% of the δ^13^C‐CH_4_ change rate of *M. sinensis* when incubated under light at 100 μmol m^−2^ s^−1^ with 4.3 ± 0.5‰ h^−1^ (*n* = 3, *p* = 0.151 compared to *M. sinensis* in the dark).

## 4 DISCUSSION

### 4.1 Limitations of mixing ratio measurements and possibilities of isotope labelling experiments

The dynamic incubation measurements conducted in this study enabled detection with high resolution nearly in real‐time of any change resulting from alteration of the experimental parameters. The application of isotope labelling and measurement techniques allowed us to observe the immediate effect on the formation of ^13^C‐CH_4_ after the application of ^13^C‐labeled DMSO when light conditions were changed during the incubation experiments. The observed changes in CH_4_ mixing ratios from our experiments suggest that considering CH_4_ mixing ratios alone would make drawing conclusions about the effects of light/dark cycles and light intensity very difficult and, in some cases, impossible. As calculated values for CH_4_ formation from ^13^C‐labeled DMSO are in the range of only a few ppbv, evaluating changes in CH_4_ mixing ratio due to environmental or physiological factors in this range are hard to resolve (Brüggemann et al., 2009; Keppler et al., 2008; Lenhart, Althoff, et al., 2015). However, the isotope labelling approach revealed considerable effects of light and light intensity on the patterns of released ^13^C‐CH_4_ and thus on CH_4_ formation by both plant species. Therefore, this technique allows to better resolve the influences of environmental and physiological factors in and between different plant species. Please note, that the applied isotope approach has only limited use for determining total non‐methanogenic CH_4_ emission rates by vegetation, as the observed changes in δ^13^C‐CH_4_ values are limited to only one potential precursor compound (DMSO). However, vegetative CH_4_ formation via other precursor compounds and pathways occurs simultaneously and is not monitored by this method. The recently proposed common mechanism of CH_4_ formation across all living organisms by Ernst et al. (2022) supports the application of ^13^Clabeled methylated compounds for tracking the formation of CH_4_. Thereby, CH_4_ is produced via the reaction of ROS, free iron, and methylated compounds within the cells of organisms.

### 4.2 Effect of dark–light cycles

The calculated increase of headspace δ^13^C‐CH_4_ change rates showed that ^13^C‐CH_4_ formation from ^13^C‐labeled DMSO when applied to leaves of *N. tabacum* and *M. sinensis* plants was related to light exposure. Moreover, the results from both dynamic and static isotope labelling incubation experiments showed similar patterns for the formation of CH_4_ when they are expressed as δ^13^C‐CH_4_ change rates. (Figure 5). During exposure to light, both plant species emitted ^13^C‐enriched CH_4_ as indicated by higher δ^13^C‐CH_4_ change rates. Interestingly, *N. tabacum* showed no change in δ^13^C‐CH_4_ values during incubation in the dark, while δ^13^C‐CH_4_ change rates by *M. sinensis* still accounted for about 50% compared to those incubated under light exposure. Furthermore, in all cases, the change in δ^13^C‐CH_4_ values was observed to occur almost immediately after the plants were exposed to light (see Figures 3 and 4). In the light–dark–light dynamic incubation experiments (Figure 4) this pattern was further characterized by increasing headspace δ^13^C‐CH_4_ change rates when plants were exposed to light which decreased again under dark conditions, indicating a change toward lower emissions of ^13^C‐CH_4_ with the latter treatment. The observed decline in δ^13^C‐CH_4_ values during incubation is caused by the continual dilution of the headspace of the incubation flasks with laboratory air (δ^13^C‐CH_4_ values used for calculations were those observed for the controls at the start of the incubation period). Interestingly, during the dynamic incubation of *N. tabacum* in the dark headspace δ^13^C‐CH_4_ values decreased with time to background air isotopic values, while under the same conditions, *M. sinensis* δ^13^C‐CH_4_ values declined considerably slower and never reached background air isotopic values. Importantly, this observation implies that in the dark no measurable formation of ^13^C‐CH_4_ from ^13^C‐labeled DMSO was observed from *N. tabacum*, while *M. sinensis* still emitted ^13^C‐CH_4_ at a ~ 50% lower rate relative to that when under light (see Table S2 for formation rates of CH_4_ from ^13^C‐labeled DMSO for each incubation flask). Our results are in good agreement with observations in which CH_4_ emissions by canola, sunflower, and chrysanthemum were not only linked to the exposure of blue light but also blue light intensity (Martel et al., 2020; Martel & Qaderi, 2017, 2019). High light radiation might lead to stress reactions in plants, due to an overload of the photoreceptors. Consequently, increased ROS levels might cause higher CH_4_ formation rates due to the reaction with methylated compounds (Ernst et al., 2022). Furthermore, higher CH_4_ emissions under light irradiation were also linked to higher concentrations of amino acid methyl groups (e.g., methionine), which in previous studies were found to be precursors of CH_4_ by the reaction with ROS (Althoff et al., 2014; Ernst et al., 2022; Han et al., 2017; Lenhart, Weber, et al., 2015). Simultaneously, ROS are accumulated in plants as a consequence of irradiance stress or adjustment to changing light conditions, due to an imbalance between light energy and the cells' capacity to use it (Mullineaux et al., 2018). Interestingly, photosynthesis was recently mentioned as the main driver of CH_4_ formation in cyanobacteria (Bižić et al., 2020).

Additionally, the different responses to light and dark conditions on the formation of CH_4_ by *N. tabacum* and *M. sinensis*, clearly shown by the formation of ^13^C‐CH_4_ by *M. sinensis* in the dark while *N. tabacum* shows no CH_4_ formation, might indicate that there is possibly more than one process of CH_4_ formation in plants depending on their physiology and metabolism (e.g., *N. tabacum* as a C_3_ plant and *M. sinensis* as a C_4_ plant). However, at present, this observation remains difficult to explain and will require further investigation.

Even though the general patterns of ^13^C‐CH_4_ formation were similar for both plants and both incubation types, the measured headspace δ^13^C‐CH_4_ change rates quantitatively differed between the two types of incubation. This might likely be caused by the differences between the dynamic and static incubations. While the dynamic incubation is a flow‐through system that is constantly in exchange with ambient air, during the static incubation the headspace is not exchanged. This difference might lead at least to partial closing of stomata or larger oxidative or abiotic stress reactions in the plant species incubated under dynamic conditions and therefore explain quantitative differences between the plant species. In addition, the changes in the partial pressures of CO_2_ and O_2_ in the static incubations are larger. Nevertheless, even though a certain degree of uncertainty remains as to the cause of the differences between the observed headspace δ^13^C‐CH_4_ change rates for both incubations, the patterns observed in our experiments are comparable and therefore allow for some mechanistic interpretation of the results.

### 4.3 Influence of light intensities

Different light intensities were also observed to influence plant CH_4_ formation by *N. tabacum* and *M. sinensis*. Please note, that light intensities used for this study (100 and 250 μmol m^−2^ s^−1^) were much lower when compared to sunlight (ca. 2000 μmol m^−2^ s^−1^). During higher light intensity incubations, a trend toward higher headspace ^13^C‐CH_4_ emissions by *N. tabacum* was detected while, contrastingly, *M. sinensis* tended toward slightly lower ^13^C‐CH_4_ emissions. Even though we do not know the causal sequence of these different responses to light intensity for either plant species, a likely factor might be the differences in ROS production during the acclimation process to different abiotic stresses (here, light intensity), as ROS are not only toxic by‐products of stress metabolism but also function as signal transduction molecules in plants (Choudhury et al., 2017). A further role could be played by the different photosynthesis types of *N. tabacum* (C_3_‐plant) and *M. sinensis* (C_4_‐plant). C_3_ plants generally have a lower limit of light saturation compared to C_4_ plants, which under higher light intensities might lead to higher oxidative stress, hence more excess energy and an increased formation of ROS and therefore explain higher ^13^C‐CH_4_ formation in *N. tabacum* compared to *M. sinensis* (Ernst et al., 2022; Martel & Qaderi, 2017; Messenger et al., 2009). Moreover, the potential for higher oxidative stress and/or abiotic stress might explain the relatively high and variable SDs within replicate samples observed at the different light intensities in both dynamic and static incubations, as individual plant reactions to these stress factors might vary strongly. The influence of different light intensities on CH_4_ formation has been reported not only for plants but also for other eukaryotes. While Klintzsch et al. (2020) showed that CH_4_ production by three marine phytoplankton species, among other factors, is strongly light intensity dependent, Ernst et al. (2022) demonstrated that increased CH_4_ formation is linked to elevated oxidative stress in bacteria, archaea, fungi, animals, and plants. This indicates that light intensity and light exposure do not only influence CH_4_ formation in plants but possibly also affect its formation in other eukaryotes on a greater scale. Therefore, light intensity, while seemingly having a lower impact than dark–light changes might still play a significant role in plant CH_4_ emissions, especially between different climate zones.

### 4.4 DMSO as a precursor of CH_4_ in plants

Our labelling experiments with ^13^C‐labeled DMSO indicated that the methyl thiol group of DMSO is a potential precursor for CH_4_ formation during plant metabolism. DMSO is among a number of compounds that contain thiomethyl groups (S‐CH_3_), such as methionine, dimethylsulfide (DMS), and methionine sulfoxide, that have been shown to act as a precursor of CH_4_ formation by plants, fungi, marine algae, and cyanobacteria and other organisms (Althoff et al., 2014; Bižić et al., 2020; Ernst et al., 2022; Klintzsch et al., 2019, 2020; Lenhart et al., 2016). The above‐mentioned thioethers (methionine, DMS) and sulfoxides (methionine sulfoxide, DMSO) can be catalyzed by nonheme iron‐oxo (IV), an active intermediate that occurs in the catalytic cycles of several biological enzymatic systems (Hohenberger et al., 2012), leading to the formation of methyl radicals from the thiomethyl group and ultimately CH_4_ (Althoff et al., 2014; Benzing et al., 2017; Ernst et al., 2022). DMSO has been detected in several plants such as the sea daisy *Wollastonia biflora*, saltmarsh grasses, and sugarcane (Dacey et al., 1987; Husband & Kiene, 2007; Otte et al., 2004; Paquet et al., 1994) and is formed by the oxidation of dimethlysulfoniumpropionate (DMSP) and/or DMS, both compounds which can be produced within plant cells (Husband & Kiene, 2007). In plants, DMSO has been shown to exhibit cryoprotective, radioprotective, osmoprotective as well as antioxidant abilities (Husband & Kiene, 2007; Lee & De Mora, 1999; Sunda et al., 2002). The antioxidant properties of DMSO lead to the formation of CH_4_ through its reaction with ROS which produces a methyl radical from the organosulfur compound (Ernst et al., 2022; Herscu‐Kluska et al., 2008; Repine et al., 1981). Furthermore, higher DMSO concentrations have been linked to plant parts that showed signs of oxidative stress such as yellowing and senescing (Husband & Kiene, 2007), thus suggesting that CH_4_ formation is a response to oxidative stress and reaction with ROS is a likely mechanism in vegetation.

## 5 CONCLUSION

This study presents an isotope labelling method for making CH_4_ formation in plants under variable environmental conditions clearly visible. This was achieved by the application of ^13^C‐labeled DMSO onto the leaves of the plant species *N. tabacum* and *M. sinensis* and the measurement of headspace δ^13^C‐CH_4_ values with a CRDS and an IRMS formed during dynamic and static incubations. Furthermore, we demonstrate that DMSO, a compound known to be ubiquitous in the environment and to occur in plants, might be a potential precursor of vegetative CH_4_ emissions. The formation of CH_4_ in this study was highly dependent on exposure to light, light intensity, and plant species. Both investigated plant species produced considerably more CH_4_ when they were exposed to light compared to in the dark. However, while *N. tabacum* showed no CH_4_ formation when incubated in the dark, *M. sinensis* still showed about 50% of CH_4_ formation when exposed to light. The effect of light intensity was smaller compared to the effect of light exposure and also differed between the two plant species. While *N. tabacum* showed slightly more CH_4_ formation at a higher light intensity, *M. sinensis* produced less CH_4_ under these conditions. Even though in recent years many studies contributed to the understanding of CH_4_ formation in aerobic environments and plants, the exact underlying mechanism(s), as well as the influence of environmental factors, are still hardly known. The method of using dynamic incubations in combination with ^13^C‐labeled compounds and in‐situ measurements with a CRDS enables the visualization of changes in mechanism or pathway‐specific plant CH_4_ formation patterns almost in real‐time. Thus, this new approach has great potential not only for investigating the mechanism(s) of CH_4_ formation, but also for any underlying physiological processes that cannot be made visible alone through measurements of CH_4_ mixing ratios. Furthermore, our study indicates the highly complex nature of non‐methanogenic emissions from vegetation and the multitude of environmental and physiological factors that control emissions. It also further highlights the still profoundly uncertain entity of CH_4_ emissions by vegetation, especially with regard to global estimations and demonstrates the need for more research on the exact mechanism(s) of CH_4_ formation in vegetation.

## CONFLICT OF INTEREST

The authors declare they have no conflict of interest.

## Supporting information


DataS 1

## Data Availability

The experimental data used in this study are available from heiDATA, which is an institutional repository for research data of the Heidelberg University (https://doi.org/10.11588/data/VJFIBD).
