# Supplementary material for: Making plant methane formation visible—Insights from application of 13C‐labeled dimethyl sulfoxide
Source: Plant Environ Interact. 2022 May 6;3(3):104–17. doi: 10.1002/pei3.10076 (PMC10168057; doi:10.1002/pei3.10076)
Supplement: Supplementary file 1 — DataS 1 [file PEI3-3-104-s001.pdf]

Supporting Information

Article title: Making plant methane formation visible – insights from application of ^{13}C-labelled dimethyl sulfoxide
Authors: Moritz Schroll, Katharina Lenhart, Steffen Greiner, Frank Keppler

The following Supporting Information is available for this article:

Table S1 Flask number, plant species and biomass of the plants used for dynamic and static incubation experiments. Light intensity used for static incubation experiment is shown in right panel.

Dynamic incubation

Flask number	Plant species	Biomass (g)
1	*N. tabacum*	1.19
3	*N. tabacum*	1.66
4	*N. tabacum*	1.71
5	*N. tabacum*	0.91
8	*N. tabacum*	0.79
2	*M. sinensis*	2.55
6	*M. sinensis*	5.55
9	*M. sinensis*	5.73
10	*M. sinensis*	6.02

Static incubation

Flask number	Plant species	Light intensity (µmol m^{-2} s^{-1})	Biomass (g)
1	*N. tabacum*	100	0.77
2	*N. tabacum*	100	1.16
3	*N. tabacum*	100	0.71
7	*N. tabacum*	-	0.59
8	*N. tabacum*	-	0.85
9	*N. tabacum*	-	0.54
4	*M. sinensis*	100	6.45
5	*M. sinensis*	100	6.77
6	*M. sinensis*	100	6.60
10	*M. sinensis*	-	4.76
11	*M. sinensis*	-	4.81
12	*M. sinensis*	-	6.89

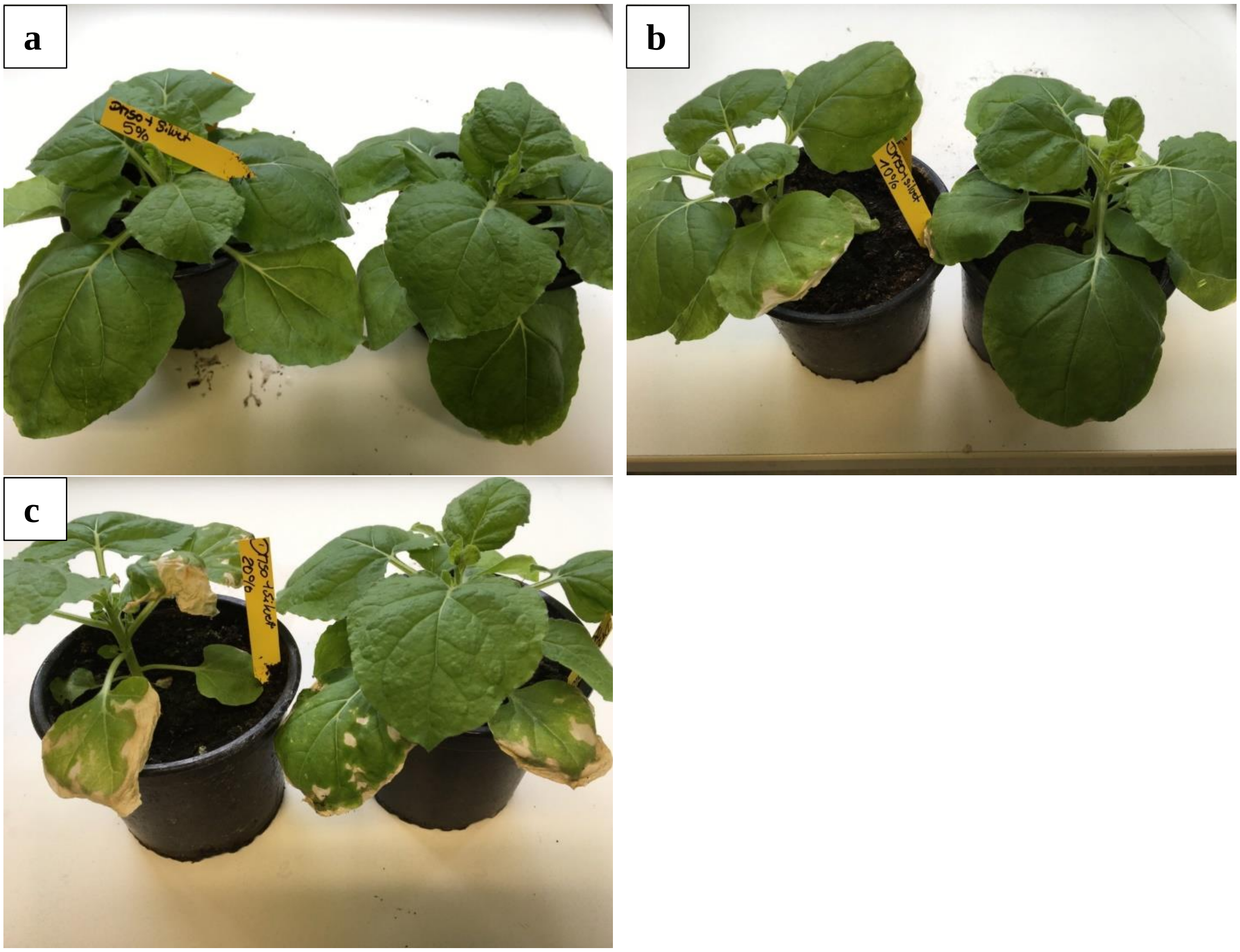

Fig. S1 Photographs of N. tabacum plants during pre-test treatment with aqueous dimethyl sulfoxide (DMSO) solutions at (**a**) 5 %, (**b**) 10 % and (**c**) 20 %.

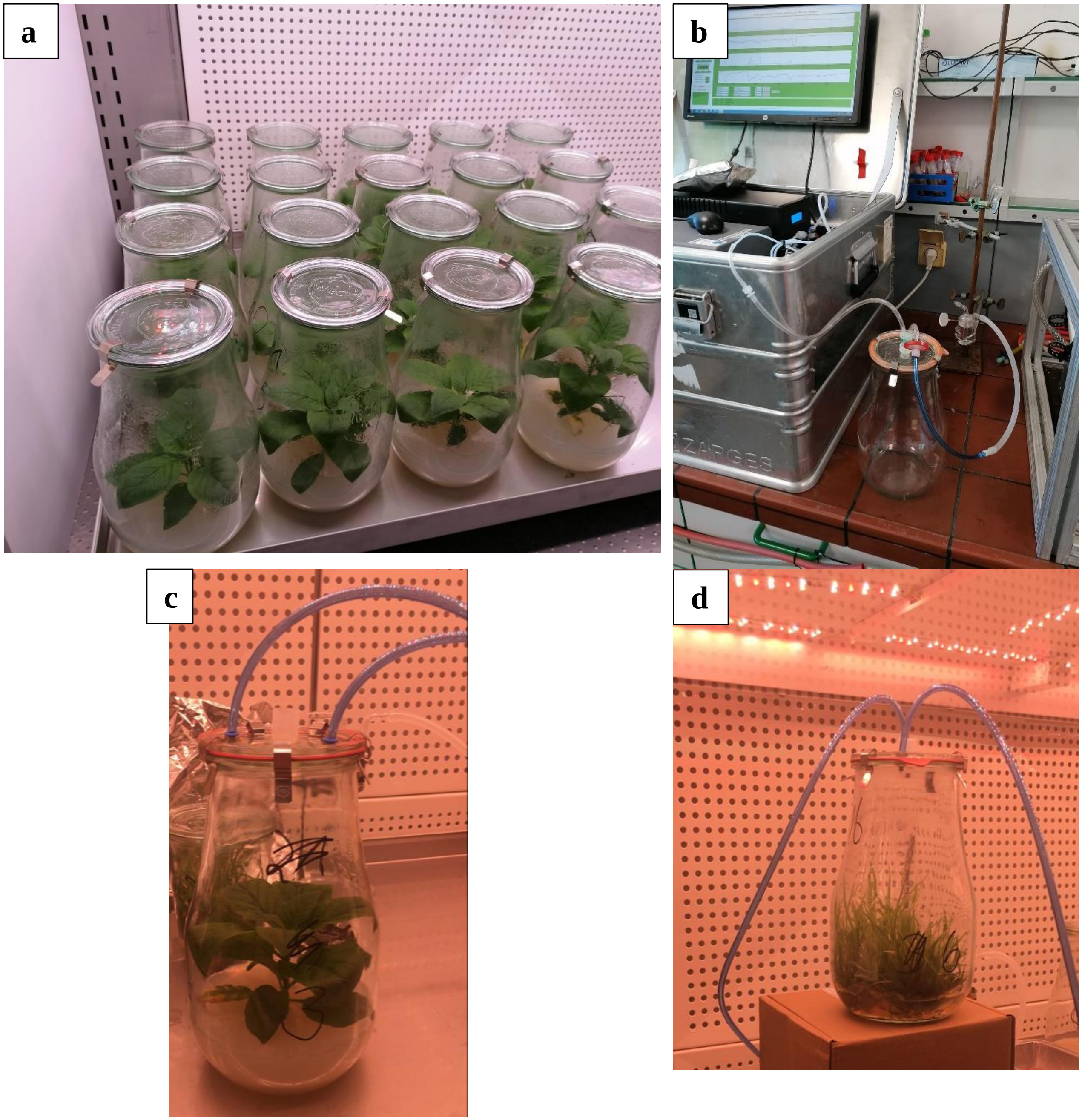

Fig. S2 (**a**) Incubation flasks containing the medium and N. tabacum plants prior to incubation, (**b**) the cavity ring-down spectroscope (CRDS) used for measurements, (**c**) *N. tabacum* and (**d**) *M. sinensis* during dynamic incubation in the climate chamber.

Method S1 Dilution model for the calculation of the $\delta^{13}C\text{-}CH_4$ change rates of the dynamic incubation experiment

In order to calculate headspace $\delta^{13}C\text{-}CH_4$ change rates in the dynamic incubation experiment, the constant dilution of the headspace gas in the incubation flask with laboratory air had to be taken into account. Therefore, two models, one for $\delta^{13}C\text{-}CH_4$ values and one for the mixing-ratio of CH_4 during the incubation were employed. The calculation of the dilution is based on a simple mass balance approach. Both models are very similar, however, for the $\delta^{13}C\text{-}CH_4$ dilution model $\delta^{13}C\text{-}CH_4$ values for the laboratory air were considered as constant with a value of -52 ‰, as it did not change significantly during the incubation period, while for the CH_4 mixing ratio model, the CH_4 mixing ratio of the most proximate laboratory air measured was used.

$$\delta^{13}CH_{4_{t(i)}} = \frac{\left\{\left[V_{flask} - (\Delta t_{inc} * Q)\right] * \delta^{13}CH_{4_{flask\ t(i-1)}}\right\} + \left(\Delta t_{inc} * Q * \delta^{13}CH_{4_{labair}}\right)}{V_{flask}} \quad (4)$$

where $\delta^{13}CH_{4t(i)}$ and $\delta^{13}CH_{4t(i-1)}$ are the stable carbon isotope values of CH_4 inside the incubation flask at the point of time t_i and t_{i-1}, $\delta^{13}CH_{4labair}$ is the stable carbon isotope value of CH_4 in the laboratory air, V_{flask} is the volume of the incubation flask, Δt_{inc} is the interval between two data points and Q is the flow rate of the CRDS.

$$c_{CH_{4_{t(i)}}} = \frac{\left\{\left[V_{flask} - (\Delta t_{inc} * Q)\right] * c_{CH4_{flask\ t(i-1)}}\right\} + \left(\Delta t_{inc} * Q * c_{CH_{4_{labair}}}\right)}{V_{flask}} \quad (5)$$

where $cCH_{4t(i)}$ and $cCH_{4t(i-1)}$ are the mixing ratios of CH_4 inside the incubation flask at the point of time ti and ti-1, $cCH_{4labair}$ is the mixing ratio of CH_4 in the laboratory air, V_{flask} is the volume of the incubation flask, Δt_{inc} is the interval between two data points and Q is the flow rate of the CRDS.

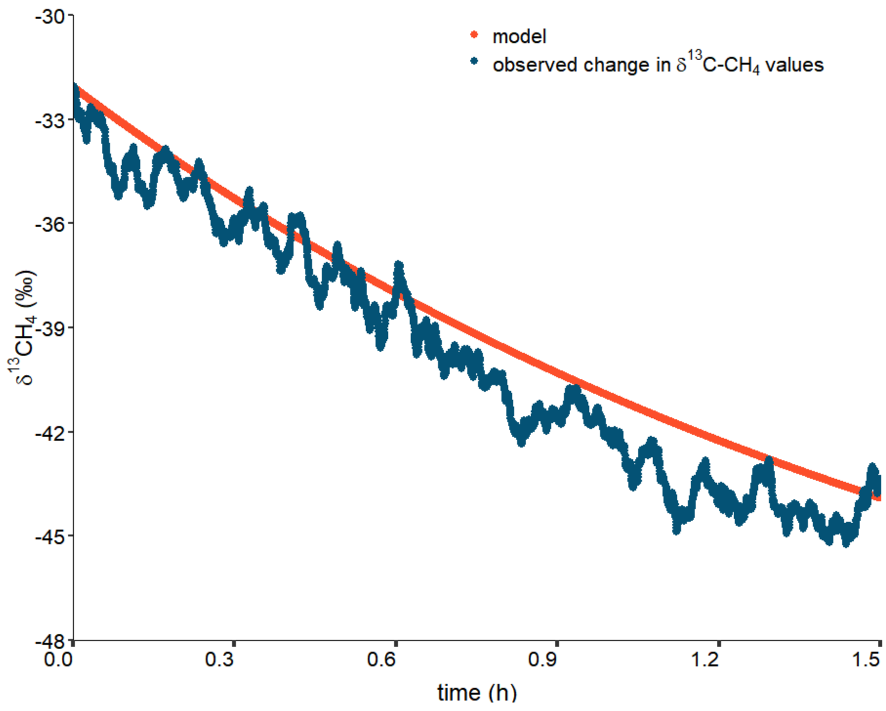

Fig. S3 Change in headspace $\delta^{13}C-CH_4$ values during dilution of headspace in the incubation flask with laboratory air. The blue line represents the data obtained during the incubation of *N. tabacum* in the dark, while the red line represents the model used in this study to correct for the dilution process during the incubations.

Method S2 Analysis of $\delta^{13}C-CH_4$ values during static incubations

The CF-IRMS was coupled to a HP 6890N GC (Agilent, Santa Clara, USA) which was directly linked to a preconcentration unit. The GC was operated with an injector temperature of 200°C, isothermal oven temperature of 30°C, split injection (10:1) and a constant carrier gas flow of 1.8 ml min^{-1} (methane-free helium). The GC was equipped with a CP-PoraPLOT Q capillary column (Varian, Palo Alto, USA) with a length of 27.5 m, an inner diameter of 0.25 mm and a film thickness 8 µm. The Delta^{PLUS}XL IRMS (ThermoQuest Finnigan, Bremen, Germany) was coupled to the GC, via an oxidation reactor and a GC Combustion III Interface (ThermoQuest Finnigan, Bremen, Germany). The oxidation reactor was operated at a temperature of 960 °C and consists of a ceramic tube, with a length of 32 mm and an inner diameter of 1.0 mm and was filled with Ni/Pt wires that were activated by oxygen. For measurement of the $\delta^{13}C-CH_4$ values, the gas samples were transferred to an evacuated sample loop (40 ml). CH_4 was trapped in the preconcentration unit by Hayesep D, separated from other compounds by GC and eventually introduced into the IRMS via an open split. High purity carbon dioxide (carbon dioxide 4.5, Messer Griesheim, Frankfurt, Germany) with a known $\delta^{13}C$ value of -23.6 ‰ (calibrated at MPI for

Biogeochemistry in Jena, Germany) was used as the working reference gas. All $\delta^{13}C$ values were normalised using two carbon dioxide reference gases of high purity (Isometric instruments, Victoria, Canada) with $\delta^{13}C$ values of -42.3 ± 0.2 ‰ and -66.4 ± 0.2 ‰ that were calibrated against IAEA and NIST reference substances.

Table S2 CH_4 formation rates from ^{13}C-labelled dimethyl sulfoxide (DMSO) by *N. tabacum* and *M. sinensis* during incubation under different light conditions.

**Plant species**	**Treatment**	**Calculated change of CH_4 mixing ratio derived from ^{13}C-labelled DMSO ($ppbv\ h^{-1}$)**
Control	light 100 $\mu mol\ m^{-2}\ s^{-1}$	0.4
Control	light 100 $\mu mol\ m^{-2}\ s^{-1}$	0
N. tabacum	dark	0
N. tabacum	dark	0
N. tabacum	dark	0.4
N. tabacum	dark	0
N. tabacum	light 100 $\mu mol\ m^{-2}\ s^{-1}$	9.4
N. tabacum	light 100 $\mu mol\ m^{-2}\ s^{-1}$	2.0
N. tabacum	light 100 $\mu mol\ m^{-2}\ s^{-1}$	0.7
N. tabacum	light 250 $\mu mol\ m^{-2}\ s^{-1}$	12.7
N. tabacum	light 250 $\mu mol\ m^{-2}\ s^{-1}$	0.5
N. tabacum	light 250 $\mu mol\ m^{-2}\ s^{-1}$	2.1
N. tabacum	light 250 $\mu mol\ m^{-2}\ s^{-1}$	4.9
M. sinensis	dark	0.9
M. sinensis	dark	5.3
M. sinensis	dark	3.6
M. sinensis	dark	2.4
M. sinensis	light 100 $\mu mol\ m^{-2}\ s^{-1}$	16.0
M. sinensis	light 100 $\mu mol\ m^{-2}\ s^{-1}$	12.1
M. sinensis	light 100 $\mu mol\ m^{-2}\ s^{-1}$	6.4
M. sinensis	light 100 $\mu mol\ m^{-2}\ s^{-1}$	6.5
M. sinensis	light 250 $\mu mol\ m^{-2}\ s^{-1}$	12.8
M. sinensis	light 250 $\mu mol\ m^{-2}\ s^{-1}$	7.5
M. sinensis	light 250 $\mu mol\ m\text{-}^{2}\ s^{-1}$	5.2

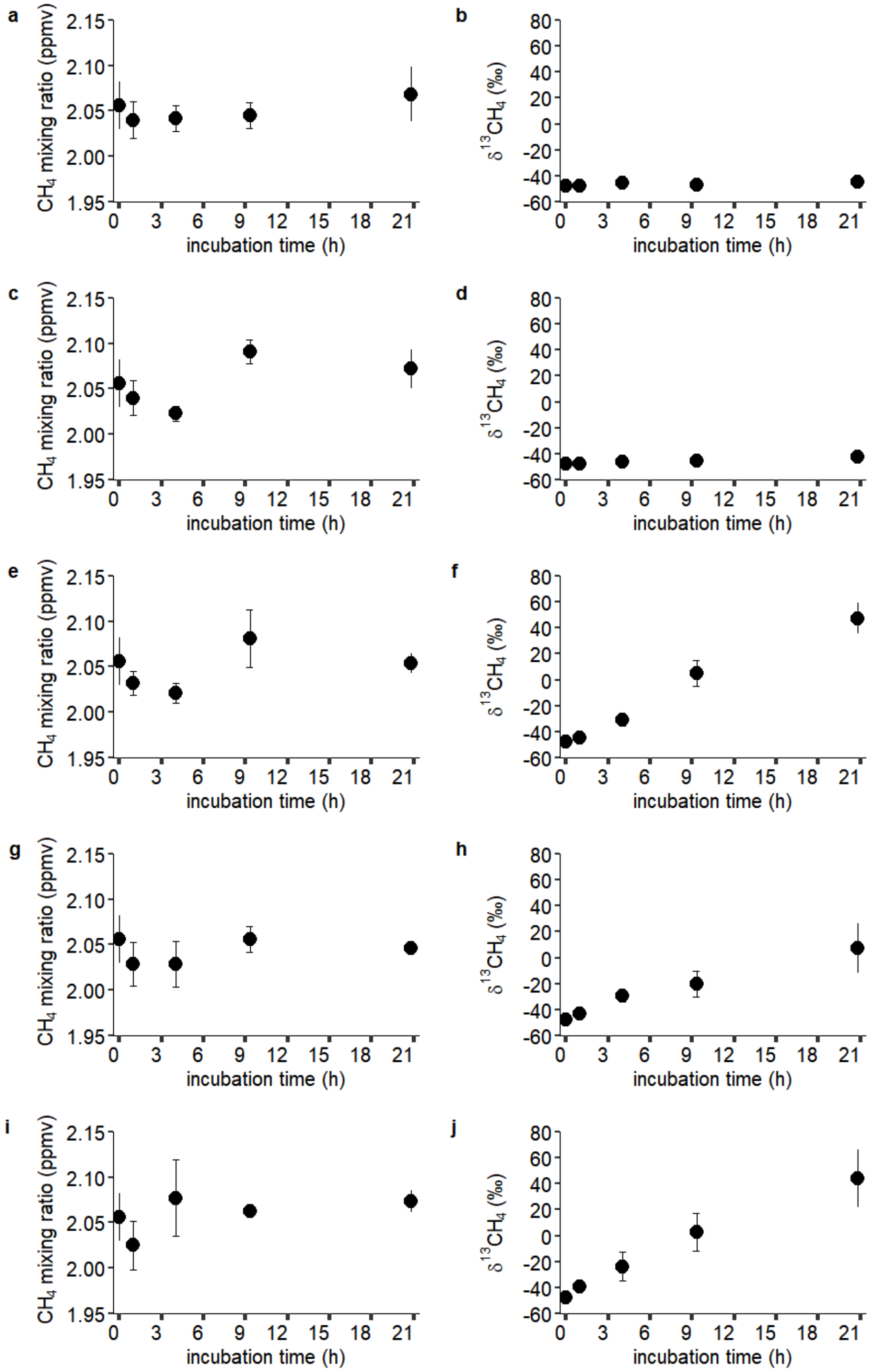

Fig. S4 Headspace CH_4 mixing ratios and $\delta^{13}C$-CH_4 values from static incubations of (**a**, **b**) control samples and *N. tabacum* (**c**, **d**) in the dark and (**e**, **f**) under light at an intensity of 100 µmol m^{-2} s^{-1} and *M. sinensis* (**g**, **h**) in the dark and (**i**, **j**) under light at an intensity of 100 µmol m^{-2} s^{-1} plants after application of ^{13}C-labelled dimethyl sulfoxide (DMSO).
